# Autoantibodies to heat shock protein 60, 70, and 90 are not altered in the anti-SARS-CoV-2 IgG-seropositive humans without or with mild symptoms

**DOI:** 10.1007/s12192-021-01215-3

**Published:** 2021-06-02

**Authors:** Jagoda Mantej, Marta Bednarek, Krzysztof Sitko, Marta Świętoń, Stefan Tukaj

**Affiliations:** Department of Molecular Biology, Faculty of Biology, University of Gdańsk, Wita Stwosza 59, 80-308 Gdańsk, Poland

**Keywords:** Heat shock proteins, Hsps, COVID-19, Severe acute respiratory syndrome corona virus 2, SARS-CoV-2, Autoimmunity, Autoantibodies

## Abstract

Highly conserved heat shock proteins (Hsps) are localized in the cytoplasm and cellular organelles, and act as molecular chaperones or proteases. Members of Hsp families are released into the extracellular milieu under both normal and stress conditions. It is hypothesized that the severe acute respiratory syndrome corona virus 2 (SARS-CoV-2) has the potential to elicit autoimmunity due to molecular mimicry between human extracellular Hsps and immunogenic proteins of the virus. To confirm the above hypothesis, levels of circulating autoantibodies directed to the key human chaperones i.e., Hsp60, Hsp70, and Hsp90 in the anti-SARS-CoV-2 IgG-seropositive participants have been evaluated. Twenty-six healthy volunteers who got two doses of the mRNA vaccine encoding the viral spike protein, anti-SARS-CoV-2 IgG-positive participants (n = 15), and healthy naïve (anti-SARS-CoV-2 IgG-negative) volunteers (n = 51) have been included in this study. We found that the serum levels of anti-Hsp60, anti-Hsp70, and anti-Hsp90 autoantibodies of the IgG, IgM, or IgA isotype remained unchanged in either the anti-COVID-19-immunized humans or the anti-SARS-CoV-2 IgG-positive participants when compared to healthy naïve volunteers, as measured by enzyme-linked immunosorbent assay. Our results showing that the humoral immune response to SARS-CoV-2 did not include the production of anti-SARS-CoV-2 antibodies that also recognized extracellular heat shock protein 60, 70, and 90 represent a partial evaluation of the autoimmunity hypothesis stated above. Further testing for cell-based immunity will be necessary to fully evaluate this hypothesis.

## Introduction

Highly conserved heat shock proteins (Hsps) are found in the cytoplasm, cellular organelles and extracellular fluids acting as molecular chaperones and proteases. Based on their molecular weight and the presence of characteristic domains, Hsps are categorized into several families, including Hsp60, Hsp70, and Hsp90 chaperones (Kampinga et al. 2009). In fact, these stress proteins can be released to the extracellular milieu and activate both the innate and adaptive immune responses (De Maio 2014; Pockley and Henderson 2018). This activation may drive to the generation of circulating anti-Hsps autoantibodies that are frequently elevated in autoimmune diseases (Tukaj and Kaminski 2019). Nevertheless, we and others have found that the anti-Hsps (auto)antibodies are also present in the serum of normal individuals (Pockley et al. 1998; Tukaj 2020).

Infections/vaccines and autoimmunity are linked fields (Cusick et al. 2012; Guimarães et al. 2015; Rojas et al. 2018). Recently, a link between COVID-19 and the development of autoimmunity has been also proposed (Cappello et al. 2020; Lucchese and Flöel 2020; Ehrenfeld et al. 2020; Kasperkiewicz 2021a, b; Hall 2021). Marino Gammazza et al. (2020) predicted that the severe acute respiratory syndrome corona virus 2 (SARS-CoV-2), the cause of the COVID-19 disease, has the potential to elicit an autoimmune reaction due to molecular mimicry between Hsps and immunogenic viral proteins. Molecular mimicry might occur when peptides derived from pathogens share amino acid sequence (linear epitopes) or structural similarities (conformational epitopes) with self-antigens. It has been found that 17 human Hsp proteins belonging to inter alia Hsp60, Hsp70, and Hsp90 chaperones shared immunogenic epitopes (at least six amino acids) with SARS-CoV-2 proteins, as analyzed by the free Immune Epitope Database and Analysis Resource (Marino Gammazza et al. 2020).

To verify the above hypothesis experimentally, levels of circulating autoantibodies directed to the key human chaperones, i.e., Hsp60, Hsp70, or Hsp90 have been evaluated in the anti-SARS-CoV-2 IgG-positive participants.

## Materials and methods

### Human blood samples

Twenty-six healthy volunteers who got two doses of the mRNA anti-COVID-19 vaccine encoding the viral spike protein (Pfizer-BioNTech COVID-19 Vaccine), anti-SARS-CoV-2 IgG-positive participants (n = 15), and healthy naïve (anti-SARS-CoV-2 IgG-negative) volunteers (n = 51) have been included in this study. Vaccinated participants were monitored for presence of anti-SARS-CoV-2 IgG to the nucleocapsid protein and the S1 domain of the viral spike protein within 3 to 5 weeks of the last dose of the vaccine. All vaccinated participants were positive to the S1 domain of the viral spike protein and additionally one person was positive to the nucleocapsid protein of the virus, as assayed by ELISA. Additional information on unvaccinated anti-SARS-CoV-2 IgG-positive participants is presented in Table 1. Serum samples were collected from blood donors from Northern Poland between December 2020 and February 2021 and stored at −20 °C until analysis. Volunteers who suffered from any (auto)immunological and skin disorders have been excluded from the study. The use of human biological material was approved by a bioethics committee at the regional medical chamber in Gdańsk (Poland) and written informed consents were performed in accordance with the Declaration of Helsinki.

**Table 1:** Characteristics of unvaccinated anti-SARS-CoV-2 IgG-positive participants

Patients’ ID no.	Anti-SARS-CoV-2 S1 (IgG) by ELISA	Anti-SARS-CoV-2 NCP (IgG) by ELISA	COVID-19 symptoms	COVID-19 verified by PCR
1	+	+	Fever, cough, fatigue, muscle and body aches, headache, loss of taste and smell	+
2	+	+	Cough	+
3	+	+	Fever, cough, fatigue, muscle and body aches	NT
4	+	+	Fever, cough, fatigue, muscle and body aches, sore throat	NT
5	+	+	Fever, fatigue, muscle and body aches	NT
6	+	+	Fever, cough, fatigue, muscle and body aches, headache,	NT
7	+	+	Fever, cough, fatigue, muscle and body aches	NT
8	+	+	Fever, fatigue	NT
9	+	+	Fever, cough, fatigue, muscle and body aches	NT
10	+	+	Loss of taste and smell	NT
11	+	+	Fever, cough, fatigue, muscle and body aches, sore throat	NT
12	+	−	Fatigue	NT
13	+	+	NR	NT
14	−	+	NR	NT
15	+	+	NR	NT

The presence of anti-SARS-CoV-2 antibodies directed to the S1 domain of the viral spike protein and/or nucleocapsid protein (NCP) were analyzed separately by commercially available anti-SARS-CoV-2 ELISA (IgG) kits. Twelve out of 15 positive volunteers reported at least one of the typical COVID-19 symptoms (e.g., fever, cough, fatigue, muscle or body aches, headache, loss of taste or smell, or sore throat) that appeared in the last 12 weeks prior to blood sampling for anti-SARS-CoV-2 IgG analysis. In two donors, the presence of SARS-CoV-2 virus was confirmed by PCR. *NR*, not reported; *NT*, not tested

### Detection of circulating anti-SARS-CoV-2 antibodies

Although SARS-CoV-2 infection is usually verified by PCR, a detection of circulating anti-SARS-CoV-2 IgG antibodies is also an accepted approach to confirm past infection in convalescents (Zhang et al. 2020). Ninety-two serum samples were screened for the presence of anti-SARS-CoV-2 antibodies directed to the S1 domain of the viral spike protein and nucleocapsid protein by a commercially available FDA-approved anti-SARS-CoV-2 ELISA (IgG) kit (EUROIMMUN, Cat. no. EI2606-9601-2 G; sensitivity: 94.4%, specificity: 99.6%) and anti-SARS-CoV-2 NCP ELISA (IgG) kit (EUROIMMUN, Cat. no. EI2606-9601-1 G; sensitivity: 94.6%, specificity: 99.8%), respectively.

### Detection of circulating anti-heat shock protein antibodies

Levels of IgG, IgM, and IgA against human Hsp60, Hsp70, and Hsp90 were evaluated in the serum samples by a home-made enzyme-linked immunosorbent assay (ELISA), as described previously (Mantej et al. 2019). Briefly, medium-binding 96-well plates (Cat. no. **504201**, Nest Scientific Biotechnology) were coated with commercially available full-length recombinant Hsp60 (Cat. no. ab78792; Abcam), Hsp90 (Cat. no. ADI-SPP-770; Enzo Life Science) or previously purified recombinant Hsp70 (Tukaj et al. 2021) proteins at a concentration of 0.5 μg/ml in 0.05 M bicarbonate buffer at 4°C overnight. The wells were blocked with 1% bovine serum albumin (BSA) in phosphate-buffered saline (PBS) at room temperature (RT) for 90 min. After washing, the evaluated sera were diluted in PBS containing 0.1% BSA (Cat. no. 05482-100G, Sigma), added to the wells and were incubated at RT for 90 min. Plates were then incubated with horseradish peroxidase (HRP)-conjugated anti-human IgG (Cat. no. 096M-4809V; Sigma)-, anti-human IgM (Cat. no. ab8507; Abcam)- or anti-human IgA (Cat. no. 41100; BioLegend)-specific secondary antibodies diluted in PBS containing 0.1% BSA at RT for 60 min. The TMB substrate solution (Cat. no. ab171523; Abcam) was used to visualize HRP enzymatic reaction and the reaction was stopped by adding H_2_SO_4_. Optical density measurements were performed at 450 nm with an ELISA plate reader (VICTOR Multilabel Plate Reader, PerkinElmer).

### Statistical analysis

Statistical analyses were performed using the GraphPad Prism (San Diego, CA, USA) software. The Shapiro-Wilk test was used to verify whether the data had normal distribution. Data was analyzed by the Kruskal-Wallis test. P values less than 0.05 were considered as significant.

## Results

### No reactivity of the anti-SARS-CoV-2-positive serum with heat shock protein 60, 70, and 90

**Fig. 1:**
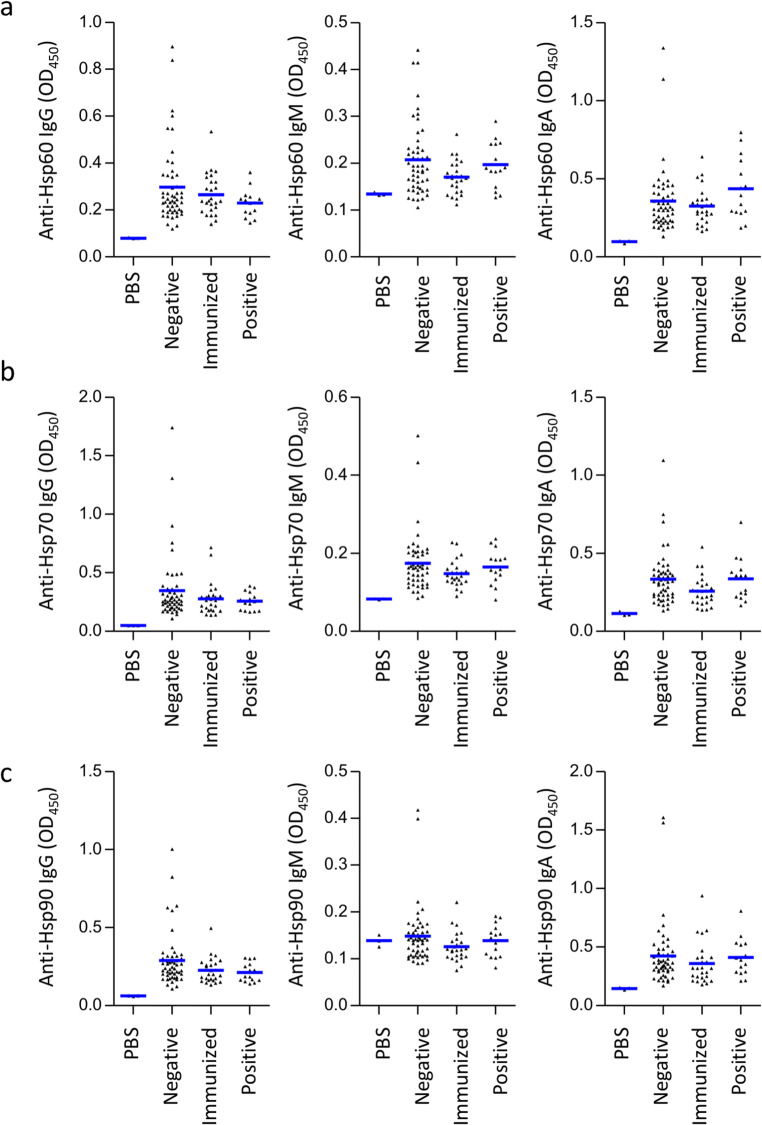
Levels of circulating anti-heat shock protein autoantibodies in the anti-SARS-CoV-2 IgG-positive patients. Levels of (**a**) anti-Hsp60, (**b**) anti-Hsp70, and (**c**) anti-Hsp90 of the IgG, IgM, and IgA autoantibody isotype in the sera of anti-COVID-19-immunized humans (n = 26), anti-SARS-CoV-2 IgG-positive participants (n = 15), and naïve (anti-SARS-CoV-2 IgG-negative) volunteers (n = 51), assessed by the enzyme-linked immunosorbent assay. Values of sera’s reactivity with the respective Hsps above the mean values of BSA reactivity (negative control) were regarded positive, as expressed by optical density measured at 450 nm (OD_450_). The dots and horizontal bars indicate individual and mean values in each group, respectively

Given the assumption that the circulating anti-SARS-CoV-2 IgG generated during either vaccination or infection might cross-react with human Hsps, we hypothesized that the healthy volunteers who got the anti-COVID-19 vaccine or the anti-SARS-CoV-2 IgG-positive participants have higher titers of anti-Hsp antibodies in their serum. Here, healthy volunteers who got two-doses of the mRNA anti-COVID-19 vaccine encoding the viral spike protein (n = 26), anti-SARS-CoV-2 IgG-positive participants (n = 15), and healthy naïve volunteers (n = 51) have been included in this study. The presence of anti-SARS-CoV-2 antibodies directed to the S1 domain of the viral spike protein and/or nucleocapsid protein were analyzed separately by commercially available anti-SARS-CoV-2 ELISA (IgG) kits (Table 1). We found that the serum levels of anti-Hsp60, anti-Hsp70, and anti-Hsp90 autoantibodies of IgG, IgM, or IgA isotype remained unchanged in either the anti-COVID-19 vaccinated volunteers or the anti-SARS-CoV-2 IgG-positive participants when compared to healthy naïve volunteers (anti-SARS-CoV-2 IgG-negative), as measured by ELISA (Fig. 1).

## Discussion

Several mechanisms have been proposed to explain the effects of viral or bacterial infections, as well as vaccines that can initiate and/or exacerbate a pathological autoimmune reaction. One such mechanism is molecular mimicry, where a foreign antigen shares sequence or structural similarities with autoantigens (Cusick et al. 2012; Guimarães et al. 2015; Rojas et al. 2018). Recently, a link between COVID-19 and the development of autoimmunity has also been proposed (Cappello et al. 2020; Lucchese and Flöel 2020; Ehrenfeld et al. 2020; Kasperkiewicz 2021a, 2021b). It is hypothesized that SARS-CoV-2 has the potential to elicit autoimmune reaction due to molecular mimicry between heat shock proteins (Hsps) and immunogenic viral proteins (Marino Gammazza et al. 2020). Hsps are a diverse group of constitutive and/or stress-induced proteins (chaperones and/or proteases) that are categorized into several families on the basis of their molecular weight and the presence of characteristic domains. Hsps mediate a range of essential cellular functions, including proper folding of polypeptides and antigen presentation (Kampinga et al. 2009; Tukaj and Kaminski 2019; Tukaj 2020). Interestingly, various Hsps might be passively or actively released from the necrotic or stressed cells, respectively (De Maio 2014; Pockley and Henderson 2018). Highly immunogenic Hsps released into the extracellular space are able to activate both the innate and adaptive immune responses and could be implicated in the autoimmune reaction (Tukaj 2020). This activation can lead to the generation of circulating anti-Hsps autoantibodies that are frequently elevated in autoimmune diseases, such as rheumatoid arthritis, coeliac disease or dermatitis herpetiformis (Tukaj et al. 2010; Kasperkiewicz et al. 2014; Tukaj et al. 2017; Mantej et al. 2019). Therefore, volunteers who suffered from any (auto)immunological and skin disorders were excluded from this study. Here, we found that the serum levels of anti-Hsp60, anti-Hsp70, and anti-Hsp90 autoantibodies remained unchanged in either the anti-COVID-19-immunized humans or the anti-SARS-CoV-2 IgG-positive participants when compared to healthy naïve volunteers. Even though proposed similarities between epitopes found in the human Hsps and the virus (Marino Gammazza et al. 2020; Cappello et al. 2020; Lucchese and Flöel 2020) were not confirmed herein, we are aware of the limitations of our approach. For example, Hsps are subject to conformational changes depending on a huge variety of factors found in vivo and under experimental conditions (Chavez et al. 2016) that might contribute to the final outcome of this study. Also, further testing for cell-based immunity, not limited to the humoral autoimmune reactions, will be necessary to fully evaluate proposed hypothesis and to investigate potential long-term consequences of such immune cross-reactivity. Finally, more advanced experimental approaches, such as epitope mapping would be necessary to find out whether the proposed shared-epitopes might be cross-recognized by anti-SARS-CoV-2 antibodies generated during infection and could drive autoimmunity via molecular mimicry.

## Conclusions

Based on our preliminary results, we can conclude that the extracellular heat shock proteins 60, 70, and 90 are not always targeted by the anti-SARS-CoV-2 antibodies raised in human serum after either infection or immunization. Further investigations with higher number of participants are needed to clarify the role of Hsps and other autoantigens in the course of the SARS-CoV-2 infection and after anti-COVID-19 vaccination.
